# Trajectories and revolutions in popular melody based on U.S. charts from 1950 to 2023

**DOI:** 10.1038/s41598-024-64571-x

**Published:** 2024-07-04

**Authors:** Madeline Hamilton, Marcus Pearce

**Affiliations:** 1 Music Cognition Lab, Queen Mary University of London, London, E1 4NS UK; 2 Department of Clinical Medicine, Aarhus University, Aarhus, Denmark

**Keywords:** Cultural evolution, Human behaviour

## Abstract

In the past century, the history of popular music has been analyzed from many different perspectives, with sociologists, musicologists and philosophers all offering distinct narratives characterizing the evolution of popular music. However, quantitative studies on this subject began only in the last decade and focused on features extracted from raw audio, which limits the scope to low-level components of music. The present study investigates the evolution of a more abstract dimension of popular music, specifically melody, using a new dataset of popular melodies spanning from 1950 to 2023. To identify "melodic revolutions", changepoint detection was applied to a multivariate time series comprising features related to the pitch and rhythmic structure of the melodies. Two major revolutions in 1975 and 2000 and one smaller revolution in 1996, characterized by significant decreases in complexity, were located. The revolutions divided the time series into three eras, which were modeled separately with autoregression, linear regression and vector autoregression. Linear regression of autoregression residuals underscored inter-feature relationships, which become stronger in post-2000 melodies. The overriding pattern emerging from these analyses shows decreasing complexity and increasing note density in popular melodies over time, especially since 2000.

## Introduction

The search for comprehensive scientific accounts of the history and development of contemporary popular musical cultures is still in its infancy. Here we examine popular music from North America and Europe as well as other culturally-related regions, with a focus on music that has reached the top of the U.S. *Billboard* music charts from 1950 to the present day. The fields of social philosophy, musicology, economics, sociology, and media studies have produced sophisticated and informed narratives throughout the twentieth century^1–6^, but the lack of digitized collections of popular music and methods for analyzing them systematically kept quantitative support for these narratives out of reach. By the early 2010s, the availability of datasets of popular lyrics, annotations, and recordings, in combination with the maturation of the field of music informatics, allowed for studies of popular music history that tested clear scientific hypotheses^7–22, 23^.

These analyses have both contributed to the discourse on contemporary popular music and transformed it. They provide much-needed evidence for and against claims espoused by earlier qualitative accounts of music history. For example, Mauch et al.^8^ analyzed audio segments of over 15,000 recordings covering most of the *Billboard* Hot 100 from 1960 to 2010 and found quantitative evidence for “revolutions” in music known to exist only informally before, such as the expansion of rock music in the mid-60s and hip-hop related genres in the 90s. However, the study also produced results that challenged some aspects of the earlier narratives, for example, the significance of the British Invasion in popular music history^8^. In the mid-1960s, the soaring popularity of rock 'n' roll in the United States has been attributed to the boom of British rock groups such as The Rolling Stones, The Kinks, and especially The Beatles, who were famously greeted by thousands of screaming fans when they visited The United States for the first time in 1964^24^. But Mauch et al. used topic analysis of song recordings from the same period to argue that the British Invasion could not have been the sole cause of rock's rising popularity in the U.S. because the topic evolution trajectory was already changing before it began. In this way, quantitative studies of popular music can supply nuanced insight to discussion of popular music history in more qualitative fields. At the same time, it seems there is now no excuse to forgo utilizing the current music datasets and music information processing technologies when forming arguments about music history; as Mauch et al. put it, “those who wish to make claims about how and when popular music changed can no longer appeal to anecdote, connoisseurship and theory unadorned by data”^8^.

Yet, the picture of contemporary popular music painted by the current scientific studies on the subject is still incomplete. Many existing studies either operate in the textual domain^7,11^, examining song lyrics, titles, and tags, or in the audio domain, extracting and then analyzing timbral or harmonic features from recordings^8–10, 12–14^. However, symbolic representations of music, i.e., musical scores or similar representations, capture different properties than audio representations and therefore can supply a different set of insights. Features of music that would have to be extracted with some effort and error in the audio domain, such as melody, onset times and lengths of notes, are encoded explicitly in the symbolic domain. This makes many of the more abstract elements which are highly salient in the perception of music, such as melody, rhythm, and tonal and metrical structure, far more accessible in a computational context if symbolic representations of music are used. Thus, though studying audio features is valuable, computational analysis in the symbolic domain is also necessary to achieve a comprehensive understanding of popular music history.

Since creating high-quality symbolic data is usually very labor-intensive, current empirical studies investigating the history of popular music in the symbolic domain exist on a much smaller scale than those in the audio or textual domains. Symbolic corpus analyses of harmony in popular music^15,16, 25, 26^ tend to use the McGill Billboard Corpus^26^, which consists of chord annotations for approximately 1300 tracks from the *Billboard* Hot 100 charts, or the Rolling Stone Corpus^25^, which contains melody and chord transcriptions of 200 of the songs on American magazine *Rolling Stone*’s “500 Greatest Songs of All Time” list. The MCFlow corpus^18^, with 124 transcriptions of rap songs, allows for rhythmic analysis of non-pitched vocals.

For popular music that is not rap, however, melody is arguably the most salient musical dimension; when asked to sing a popular song, people will usually sing its melody, as opposed to its bassline or drum pattern. Previous empirical studies investigating the history of popular music in the symbolic domain have found an increase in event density, or the number of note events per second or per bar, as well as increases in phrase repetition and syncopation^19–21^. However, the quality and size of the corpora analyzed in these studies greatly limit their statistical power and ability to construct a detailed narrative of popular music history. For example, the Pop20c corpus^19^ consists of transcriptions of the most popular song of each year from 1900 to 1999, or 100 songs. In^20^, the pre-millennial portion of the Rolling Stone corpus is compared to a corpus of melodic transcriptions of 25 songs released post-2000, which only amounts to 225 songs. The authors of^21^ use automatic transcription methods to create about 1500 transcriptions, but they acknowledge that the data is “messy” and that this may affect the validity of the results. Therefore, analyses of larger, yet still high-quality, corpora are needed to study the history of popular music in more detail and assess the strength of the conclusions reached by the previous studies.

To this end, we use the new Billboard Melodic Music Dataset (BiMMuDa) to investigate the history of contemporary popular melody. The BiMMuDa is a dataset of over 1000 MIDI files containing the main (vocal) melodies from the top 5 songs of each year from 1950 to 2022, according to the *Billboard* year-end singles charts. With transcriptions from 366 songs, it is the largest high-quality corpus of melodies in the symbolic domain, larger than the notable CoCoPops dataset^27^, which has melodic transcriptions of 214 of the songs annotated in the McGill Billboard Corpus. The BiMMuDa’s size and quality make it capable of serving as an adequate sample for the scientific study of contemporary popular melody. With it, we can identify trajectories in popular melody and compare them with those found in earlier accounts. Does popular melody have distinct revolutions, or do its turning points correspond to previously found revolutions within timbre, harmony, and genre? How are revolutions in popular melody characterized in terms of melodic features?

## Results

### Overview

To answer these research questions, we performed a time series analysis on a feature set characterizing historical popular melody. The dataset was compiled via manual transcription of melodies into MIDI format. The features used can be conceived as characterizing the complexity of a melody as it would appear to a listener. Perceived complexity has been related to the number and diversity of pitches and durations making up a melody, as well as measures of how clearly a melody implies a tonal or metrical interpretation^28^. Various measures of complexity were computed for each melody and then averaged by year to create a multivariate time series. Change point detection was applied to identify structural breaks in the time series, or “melodic revolutions”. These changepoints divided the time series into segments, or “eras”. Autoregression was performed on each time series for each era separately. The residuals of each autoregressive model were then regressed against the other features to identify inter-feature relationships. Finally, a vector autoregressive model was fitted to each era for multivariate time series modeling.

### Melody transcription

The Billboard Melodic Music Dataset is a collection of 1131 MIDI files containing the main melodies from the top 5 songs of every year according to the Billboard year-end singles charts from 1950 to 2023. In this context, we define “melody” to be a sequence of non-overlapping pitched events played with a particular rhythm (defined by the inter-onset interval between each event), and “main melody” as the most prominent melody in the piece, which is almost always the vocal melody. Melodies are separated into different files by section (e.g., verse, chorus); most songs have 2–4 melodies. The average melody length is 22.74 s (SD = 9.67), and the average number of note events is 48.63 (SD = 24.20). For complete information about the dataset, including the transcription process, available metadata, and summary statistics, see Hamilton et al.^29^.

### Feature selection

**Figure 1:**
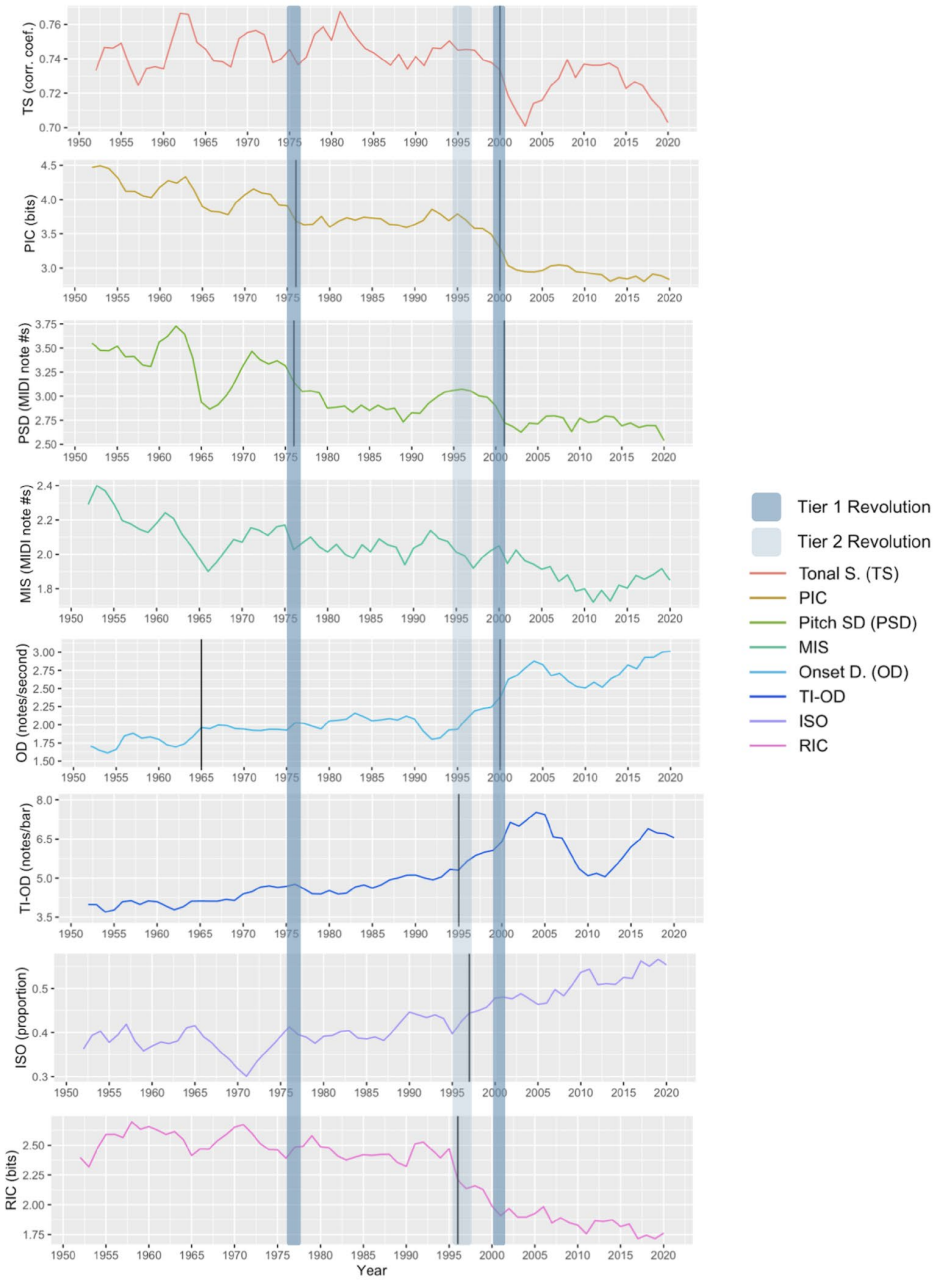
The eight time series comprising the feature set with the changepoints detected by the univariate and multivariate methods. Changepoints detected by the univariate method are represented with solid black lines. Periods identified as revolutions by both methods (Tier 1 revolutions) have strong evidence and are highlighted in dark blue. Periods identified as revolutions by only one method (Tier 2 revolutions) have moderate evidence and are highlighted in light blue. All time series data has been smoothed with a two-year averaging window forwards and backwards, so values begin at 1952 and end at 2020.

Complexity can be captured either in terms of features (e.g., pitch range, pitch interval size, note density, duration variability) or in information-theoretic terms given a statistical analysis of the pitch and timing of the notes in a melody^28^. Here we combine both approaches aiming for broad (though not necessarily exhaustive) coverage of different complexity measures. Several features were calculated per melody, from basic feature-based descriptors to more sophisticated information-theoretic measures of complexity^30^ which have been shown to predict complexity perception for both pitch and rhythm^31–33^. An initial set of 16 features was selected to cover a wide range of melodic characteristics (see supplementary materials). Features were discarded if the absolute value of their correlation coefficient with another feature was above 0.9. The resulting set contains eight features, outlined in Table 1. Four features characterize the melody's pitch content and four characterize the melody's rhythmic content. Features were averaged per year to create a multivariate time series; see Fig. 1. Time series values for 2023 were excluded from analysis so they could serve as a small test set for forecasting (see supplementary materials).

**Table 1:** Descriptions of the eight features selected for analysis (see the supplementary materials for more detailed definitions).

Feature	Type	Description
Tonal strength	Pitch	Degree of conformity to one of the 24 Western musical keys
Pitch information content (PIC)	Pitch	Information-theoretic unpredictability of the melody’s pitches
Pitch standard deviation	Pitch	Standard deviation of the melody’s pitches
Melodic interval size (MIS)	Pitch	Average distance between consecutive pitches
Onset density	Rhythm	Average number of notes per second
Tempo-invariant onset density (TI-OD)	Rhythm	Average number of notes per bar
Isochrony proportion (ISO)	Rhythm	Proportion of pairs of consecutive inter-onset intervals that are equal
Rhythmic information content (RIC)	Rhythm	Information-theoretic unpredictability of the melody’s rhythmic structure

### Changepoint detection

Changepoint detection algorithms identify significant shifts in the mean and variance of time series. In this context, changepoint detection can uncover melodic revolutions and when they occur. Two methods were used to select changepoints:

1. *Aggregate univariate changepoint detection—*changepoint detection was performed on each feature individually with four different detection algorithms (see the supplementary materials for details). If a specific time point was identified as a changepoint for at least two features, it was accepted as a melodic revolution.
2. *Multivariate changepoint detection—*multivariate changepoint detection provides the detection algorithm with all features simultaneously so that detection is holistic. Four different multivariate algorithms were applied to ensure robust results. See the supplementary materials for details on the algorithms used.

The revolutions for which there is the strongest evidence are those uncovered by both changepoint selection methods; these are referred to as “Tier 1” revolutions. Revolutions uncovered by only one method are referred to as “Tier 2” revolutions. In the univariate method, and when comparing results between the two methods, we allowed slight deviations from a median changepoint (see supplementary materials for details).

Figure 1 summarizes the outcomes of both methods. In the univariate method, changepoint detection identified 1–2 changepoints for all features except the MIS feature. The univariate method recognized 1976, 1995–1997 and 2000–2001 as periods of significant change, while the multivariate method identified 1975 and 2000 as changepoints. Thus, the Tier 1 revolutions are 1975–1976 and 2000–2001 (henceforth these will be abbreviated as 1975 and 2000, respectively), and 1995–1997 (henceforth abbreviated as 1996) is a Tier 2 revolution.

The Tier 1 revolutions divide BiMMuDa's coverage into three eras: 1950–1974, 1975–1999, and 2000–2022. In the first era, 1950–1974, melodies have relatively high pitch-related (PIC) and rhythmic (RIC) information-theoretic complexity, an average pitch interval of about 2.3 semitones (with two-thirds of pitches lying within an approximately 7-semitone range), about 1.8 notes per second, and 4.2 notes per bar. Second-era melodies (1975–1999) have lower pitch-related and rhythmic complexity, an average pitch interval of about 2.1 semitones, about 2 notes per second, and 5 notes per bar. Two-thirds of pitches lie within a 6-semitone range. Finally, in the third era from 2000 onward, melodies have the lowest pitch-related and rhythmic complexity, with an average pitch interval of about 2.0 semitones, two-thirds of pitches lying within a 5.5-semitone range, 2.8 notes per second, and 6.3 notes per bar. Both revolutions are characterized by decreases in rhythmic and pitch-related information-theoretic complexity; the second revolution is also marked by a significant increase in Onset Density.

### Autoregression and regression of residuals

Since the time series exhibit significant autocorrelation (current values can be predicted by a linear combination of previous values), autoregressive models are appropriate for describing their behavior. Additionally, when exploring relationships between time series, it is helpful to first partial out autoregressive behavior as it ensures that linear regressions are statistically valid. Autoregressive models were fit to the time series for each era, with an era-dependent maximum lag to encourage parsimony. The maximum lag for each era was set to be approximately 20% of the number of observations in the era, which yielded maximum lags of 5, 5 and 4 for Eras 1, 2 and 3, respectively. Within these restrictions, the model with the best nRMSE was selected. Goodness-of-fit values for each autoregressive model are given in Table 2. From the table, it is clear that the time series values can be partially predicted from previous values regardless of era, though there were still significant residuals.

**Table 2:** Goodness-of-fit values and lags of the fitted AR models for each era and feature.

Feature	Era 1	Era 2	Era 3
Tonal strength	0.20 (5)	0.14 (4)	0.19 (4)
PIC	0.15 (5)	0.15 (5)	0.11 (2)
Pitch SD	0.22 (3)	0.10 (4)	0.16 (3)
MIS	0.14 (3)	0.18 (4)	0.13 (1)
Onset density	0.17 (5)	0.20 (4)	0.15 (4)
TI-OD	0.15 (5)	0.11 (4)	0.13 (4)
ISO	0.19 (5)	0.23 (5)	0.21 (1)
RIC	0.16 (5)	0.21 (4)	0.20 (1)

RMSE values are normalized by the range of the original feature values. Note that an autoregressive model of lag *n* cannot give predictions for the first *n* time positions; these are ignored when computing goodness-of-fit.

To test for relationships between features, the residuals from the autoregressive model of one feature can be regressed against the values of other features. For each era, this was done for all 56 (8 features times 7 possible predictors) combinations of features. In cases where a residual had more than one significant predictor, multilinear regression could not be performed due to collinearity between predictors. The results are displayed in Table 3. In Era 1, there is an inverse relationship between Onset Density and MIS: melodies with larger melodic intervals have fewer notes per second, on average. Additionally, melodies with more notes per second are less isochronous in Era 1. This relationship reverses in Era 3, where ISO and Onset Density are positively related. In Era 2, the only significant inter-feature relationship is between PIC and Onset Density: melodies with higher pitch-related complexity tend to have fewer notes per second. This relationship holds in Era 3.

**Table 3:** Results of regressing the autoregression residuals of each feature on the other features.

Era	Dependent	Independent	Estimate	p-value	R-squared
1	Onset density	MIS	− 0.123	0.0018	0.302
1	ISO	Onset density	− 0.369	0.026	0.272
2	Onset density	PIC	− 0.409	0.017	0.263
3	Tonal S	Pitch SD	1.18	0.0025	0.466
3	Pitch SD	Tonal S	0.183	0.0083	0.361
3	Onset density	ISO	0.298	0.0045	0.427
3	Onset density	PIC	− 0.664	0.028	0.283
3	Onset density	RIC	− 0.558	< 0.001	0.576
3	ISO	RIC	− 0.461	0.014	0.292
3	RIC	ISO	− 0.305	0.0026	0.404

Era 3 yielded the largest number of significant inter-feature relationships and the relationships with the highest R-squared values. Tonal Strength and Pitch SD are positively related: melodies which adhere more strictly to the relevant key’s pitch classes have a larger spread of pitches, and vice versa. Onset Density exhibits an inverse relationship with both PIC and RIC; melodies with lower pitch-related or rhythmic complexity values have more notes per second. Finally, ISO and RIC are inversely related, with more rhythmically complex melodies being less isochronous and vice versa.

### Vector autoregression

**Figure 2:**
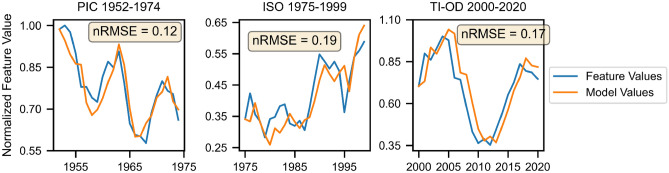
Original vs. VAR-fitted values for the PIC (Era 1), ISO (Era 2) and TI-OD (Era 3) features, with nRMSE values for each fit.

Vector autoregression (VAR) models relationships between time series simultaneously by representing the current value of a feature as a linear combination of previous values of itself as well as previous values of the other features. Thus, though less interpretable, they have greater predictive power than the individual autoregressions and regressions. For each era, a VAR model was fit to the feature set (see the supplementary materials for more information). The three best fits are illustrated in Fig. 2. The Era 1 VAR produces the best accuracy measure, modeling PIC with an nRMSE of 0.12 The Era 2 VAR models the ISO feature well (nRMSE = 0.19), and the Era 3 VAR models TI-OD with an nRMSE of 0.17. See the supplementary materials for details on forecasting 2023 feature values.

## Discussion

Our analysis presents strong evidence for two melodic revolutions in the history of popular music: one in 1975 and another in 2000. In addition, there is moderate evidence for a revolution in 1996. Regression revealed an increase in inter-feature relationship strength after the year 2000, after partialling out autoregressive behavior.

The melodic revolutions revealed by change point detection only partially coincide with those identified by Mauch et al.'s study in^8^, which conceptualized songs as distributions of harmonic and timbral “topics” and examined their frequencies over time. Their analysis located revolutions in 1966, 1983, and 1991, as opposed to the “stronger” revolutions in 1975 and 2000 and “weaker” revolution in 1996 identified by the present study. The 1975 melodic revolution is perhaps a manifestation of the rise of the new wave, disco and stadium rock genres, but according to Mauch et al., a timbral/harmonic revolution does not appear until seven years later, in the early 1980's. Mauch et al. also label 1991 as the simultaneous decline of the stadium rock era and the ascent of the hip hop era. Meanwhile, according to our analysis the surge of hip-hop melody does not occur until 1996, or perhaps even later around the year 2000.

The revolutions identified in our analysis do not necessarily conflict with those in^8^; revolutions in melody do not have to accompany revolutions in timbre or harmony and vice versa. Alternatively, the discordance may be due to a difference in coverage between the data used in this study and the data used in Mauch et al.'s study. Mauch et al.'s dataset is much larger than the BiMMuDa: it covers almost all of the weekly Billboard Hot 100, while the BiMMuDa represents only the top five songs of each year. Wider coverage may allow for early detection of revolutions, since a newly popular genre may inhabit the lower ranks of the Billboard Hot 100 for several years before it rises to the very top positions. This could be why the final revolution identified in Mauch et al.'s study is earlier than the final revolutions detected in this study.

However, it is also important to note that revolutions occurring in the same period do not necessarily have the same historical cause. Mauch et al. characterize their 1991 revolution as the ascent of hip-hop, but the songs represented in the BiMMuDa in the 1990s do not heavily feature the genre; only about 15% of the dataset’s songs between 1990 and 2000 are hip-hop. It is possible that hip-hop's rising popularity in the 1990s influenced the melodies of other genres, leading to the 1996 and 2000 revolutions identified in this paper, but these two revolutions could also be caused by unrelated phenomena. For example, major music studios adopted digital audio workstations (DAWs) as their primary music-making apparatuses in the early 1990s^34^. DAWs are fundamentally loop-based, which could implicitly prompt users to repeat melodic phrases more often, leading to the melodic revolution in 1995 pinpointed in this study. Nevertheless, determining the historical causes of these revolutions is outside the scope of the current study; investigation of these questions with thorough hypothesis testing is needed.

Concerning overall trajectories, the unidirectionality of melodic features deserves comment. Both popular^35,36^ and academic^37,38^ theories of fashion, film and music emphasize cycles powered by nostalgia, where genres and styles periodically ascend, decay, and resurface in modified form. In line with these theories, some features of the BiMMuDa, such as BPM, do indeed exhibit cyclic behavior over time (see the supplementary materials). This does not seem to be true with melody, at least over the seven decades included in the analysis. In the current study, melodic features move in a single direction, either increasing or decreasing steadily since 1950. The number of notes per second in melodies has increased dramatically, while markers of both pitch and rhythm-related complexity in melody have experienced mostly undisturbed decreases. The only exception is tempo-invariant onset density, which exhibits some cyclical behavior after the year 2000. But in general, identified melodic revolutions, rather than reversing the trajectories of these features, simply accelerate movement; in fact, the term “revolution” may not even be an apt descriptor. Whether a “true” melodic revolution, in which the direction of feature trajectories is reversed, will occur in the next decade is an interesting question that can be answered scientifically so long as the BiMMuDa is continuously updated.

When discussing the implications of this study, two points must be made. First, the study is incapable of assigning value judgements to the music it analyzes. To attribute these findings to more recent music being “bad” or to its listeners having “bad taste” would be to go beyond the scientific evidence into the realms of subjective opinion. In empirical aesthetics, the complexity of a musical piece is not positively linearly related to the pleasure it elicits; rather, listeners most prefer pieces of intermediate complexity according to Berlyne’s inverted-U theory^39^. Additionally, a decrease in the complexity of melodies does not suggest a decrease in the complexity of other musical components. Timbre and harmony, for example, can be rich sources of complexity in music, and evidence from other studies indicate that complexity along these dimensions has *not* decreased from 1960 to 2010^8^. Second, the size of the dataset used in the study is a critical limitation which severely curbs the breadth of its implications. Because the sample includes only the five most popular songs of each year, it cannot be said to represent U.S. or Western popular music more generally. Therefore, we emphasize that this is a preliminary study with restricted statistical power, and a much larger dataset of melodies is needed to verify the study’s conclusions. The labor-intensive process of manual transcription prevented the use of a larger sample for the current study, but in the future, the dataset could be expanded to include, for example, melodies from the top ten songs of each year, or it could be combined with the currently existing datasets. Still, the dataset used in this study is still the largest dataset of manually transcribed melodies to date, and the current study helps substantiate conclusions made by smaller corpus studies, such as the claims of increase in repetition and event density in popular melodies made in^20,21^.

Finally, we focus on the question of causation, that is, *why* are the melodies of the most popular songs increasingly repetitive, simple, isochronous, and dense? Why have these kinds of melodies come to be preferred? Any answer ventured here is purely speculative, as the current work is incapable of offering any conclusive evidence concerning causation. With this in mind, we offer two categories of potential explanations.

### Timbre and onset density as new carriers of musical complexity

David Temperley's theory of communicative pressure states that increased complexity in one aspect of music necessitates increased stability in other aspects in order for the music to remain interpretable by the listener^40^. Temperley argues that the maintenance of this equilibrium is a driving force in the evolution of musical styles: for example, the keeping of strict tempo in rock music allows it to feature pleasant syncopation, and repeating left-hand patterns in Romantic piano music provide the regularity needed for listeners to enjoy the music's *rubato*. Thus, one possible explanation for the decrease in the complexity of melodies in popular music is that it is a response to increasing complexity along other musical dimensions. Though the present study cannot definitively establish which dimensions these are, onset density is an obvious candidate, especially for post-2000 popular melodies. In addition to imposing physical limitations on the melodies vocalists and audiences can realistically sing (large pitch intervals, for example, would be difficult to sing if there are many notes per second), increasing onset density may also be restricting other forms of complexity in melodies per the theory of communicative pressure. That is, a vocal melody with a high onset density may avoid being psychologically overwhelming to listeners only if other aspects of the melody are simple, e.g., the melody features small pitch intervals, a limited range of pitches, and lots of repetition.

Alternatively, musical complexity in popular music may be shifting away from melody altogether, instead manifesting in other aspects of the music. In particular, it is possible that timbre is increasingly carrying the complexity of today's popular music due to the expanding availability of digital instruments. In the 50s, the range of possible timbres for music production was limited to whatever sounds one could make with the physical instruments and accessories available at the time. Today, with the accessibility of digital music production software and libraries of millions of samples and loops, anyone with a laptop and an Internet connection can create any sound they can imagine. Meanwhile, pop melody remains mostly restricted to the Western 12-tone scale, at least for now. Perhaps the much roomier space of timbral possibilities is now better suited as an outlet for creativity and expression than melody, causing the focus of composition to gradually shift away from the creation of interesting melodies and towards the creation of interesting timbres. Mauch et al.'s study partially corroborates this hypothesis; they found that musical diversity in terms of timbre and harmony reached a minimum in the 80s and peaked in the early 2000's^8^.

### Fragmentation and instability in the digital age

The decrease in the complexity of melodies may be associated with aspects of the modern predicament. Film director and composer Yuval Shrem argues that linguistic and musical trends are reflections of one another^41^. The digital era increasingly demands the compression of language, so that what we have to say will stay under character limits and fit into headlines. According to Shrem, this decreases the complexity of our messages and perhaps even our ability to digest complex ideas, and this manifests in the popular music we enjoy. Philosopher and music critic Mark Fisher argues that, in addition to this fragmentation of language, we are overwhelmed with the rapid pace of modern culture and technological progress and therefore do not have the mental capacity to enjoy or create truly complex or novel art^42^.

These hypotheses remain to be tested scientifically. The expansion of the BiMMuDa to increase its coverage of the year-end singles charts or its combination with other kinds of data may increase the dataset's ability to engage with these theories and yield analyses of higher precision.

### Supplementary Information

Supplementary Information.

## Data Availability

All code and data used in the analyses, as well as additional information about BiMMuDa, can be found at https://github.com/madelinehamilton/TAR.
