# Supplementary material for: Trajectories and revolutions in popular melody based on U.S. charts from 1950 to 2023
Source: Sci Rep. 2024 Jul 4;14:14749. doi: 10.1038/s41598-024-64571-x (PMC11224395; doi:10.1038/s41598-024-64571-x)
Supplement: Supplementary file 1 — Supplementary Information. [file 41598_2024_64571_MOESM1_ESM.pdf]

Supplementary Materials

Method Details

Feature Selection

The goal of the feature selection process was to choose a set of features that describes as many facets of melody as possible while avoiding redundancy. A preliminary set of features was compiled and computed for each melody. In cases where the absolute value of the correlation coefficient between two features was larger than .9, one feature was excluded. The choice of feature to retain depended on the prevalence of the feature in previous music research. Features were selected in this manner rather than with a dimensionality reduction method such as principal component analysis or independent component analysis in order to avoid difficulties with interpretability. Table S1 provides details about each selected feature. Table S2 lists features that were considered but ultimately rejected for this analysis.

The Information Dynamics of Music Model

Since two of the selected features utilize the IDyOM model (*2*), it is worth outlining it. Please note that the summary below is only detailed enough to provide an understanding of what the PIC and RIC features represent; it simplifies some of the model's processes and omits discussion of many of its components. See (*2*) for a more detailed explanation of IDyOM.

IDyOM (available at https://github.com/mtpearce/idyom) is a variable-order Markov model that simulates auditory expectations (*4*). At a given point in an input piece, IDyOM estimates

Table 1: Selected Feature Details

**Feature**	**Units**	**Range**	**Details**
Tonal Strength	Pearson's *r*	0.407 - 0.982	The absolute value of the largest correlation coefficient outputted by the Krumhansl-Schmuckler key-finding algorithm (*1*). The algorithm takes a 24-dimensional vector of total durations for each note in a piece and correlates it with 24 tone profiles representing the 24 Western musical keys. The tone profile with the largest correlation coefficient is the estimated key of the piece, with the value of the correlation coefficient representing the degree to which the piece conforms to the estimated key.
Pitch Information Content (PIC)	bits	0.285 - 6.04	The average information content of a melody according to IDyOM, a probabilistic model of auditory expectation (*2*). Only the short-term version of the model was used: for each melody, IDyOM calculated the probability of each note conditional upon the melody's previous notes. IDyOM was configured to predict the `pitch` viewpoint with the `interval` ⊗ `scale-degree` viewpoint.
Pitch Standard Deviation	semitones	0.00 - 9.72	The standard deviation of a list containing the MIDI note numbers of each note in the melody.
Melodic Interval Size (MIS)	semitones	0.00 - 5.40	The average pitch distance between consecutive notes. For every pair of consecutive notes in the melody, the absolute value of the difference between their MIDI note numbers is computed, and then the mean of these differences is taken.
Onset Density	notes/second	0.443 - 5.69	average number of notes per second in the melody.
Tempo-Invariant Onset Density	notes/bar	0.571 - 14.1	average number of notes per bar in the melody.
Isochrony Proportion (ISO)	none	0.00 - 1.00	the proportion of consecutive inter-onset intervals that are equal in the melody (*3*).
Rhythmic Information Content (RIC)	bits	0.234 - 7.06	The average information content of the note timings in a melody according to IDyOM, configured to use the short-term model only and set to predict the `onset` viewpoint with the `position-in-bar` ⊗ `bar-length` viewpoint.

the probability of the next note, conditional on the preceding context. It can be configured to use either or both of a short-term model (STM), which learns each melody incrementally from an initially empty state, and a long-term model (LTM), which has prior training on a large

corpus of music in a given style. The STM simulates learning of repeated patterns within a piece of music while the LTM simulates learning of stylistic patterns across multiple pieces of music. In the present research, only the STM is used to compute PIC and RIC. The probability distribution is computed by combining together predictions from models of different orders (or context length): a zeroth-order distribution only considers the marginal probability of each event, a first-order distribution considers the probability of an event conditioned on the previous event, etc. The maximum order is chosen adaptively with each event, and a smoothing technique combines the different-ordered models to produce a single distribution (*5, 6*).

A common application of IDyOM is to compute probability distributions in this manner sequentially for every event in a piece and summarize them with information-theoretic quantities, such as information content (IC) - the negative log (base 2) probability. The information content of a musical event can be interpreted as its perceived unexpectedness according to the predictive probability distribution generated by the model (*7–10*) (see also Chapter 6 in (*11*)). Thus, the events constituting a phrase that has been repeated multiple times before in the piece will have low information content, while those constituting a new phrase will have high information content. The IC values for every musical event are averaged to obtain a predictability value for the entire piece.

How does IDyOM define a "musical event"? IDyOM's multiple-viewpoint system allows for different representations of music, each of which can be used for prediction by the model independently or in combination with other representations. The two main target viewpoints, i.e., targets for prediction, are `pitch`, which represent pieces as sequences of MIDI pitch note numbers, and `onset`, which represent pieces as sequences of note onset times. IDyOM can be configured to predict pitch, onset time, or both. The same viewpoints can serve as the input representations, or source viewpoints, from which IDyOM generates predictions, but usually more musically significant representations derived from the basic viewpoints are used. For

PIC, the `scale-degree` and `interval` viewpoints are used in combination (i.e., a linked viewpoint that predicts pairs of scale degree and interval values) to predict `pitch`, thus the feature encapsulates complexity related to a melody's pitches. For RIC, `position-in-bar`, `ioi-ratio` and `bar-length` predict `onset`, so the feature represents both rhythmic and metrical complexity.

Changepoint Detection

The code for all changepoint detection and regression analyses discussed in this paper, as well as the BiMMuDa and its metadata, can be found at https://github.com/madelinehamilton/TAR.

For robust changepoint detection, four detection algorithms were applied to the time series, with each algorithm belonging to a different class of method according to the framework laid out in (*14*). 60 parameter settings were used for each algorithm to obtain 240 total function calls, or "tallies". Tallies from consecutive years were aggregated. Changepoints with half the number of possible tallies (120) were considered "true" changepoints. The algorithms were applied to both the individual and multivariate time series. "True" changepoints which appear for both the individual and multivariate time series are considered "Tier 1" changepoints, or changepoints for which there is strong evidence, while "true" changepoints which appear in more than one individual time series, but not in the multivariate time series, are considered "Tier 2" changepoints, or changepoints for which there is moderate evidence.

In their review of offline changepoint detection methods, Truong et al. (*14*) outline four approaches to changepoint detection. One method was selected from each category to use for analysis in order to minimize bias that may arise from utilizing methods from only one class. The four categories are listed below, with the individual method selected from the category in parentheses

- *Top-down (E-divisive)* - top-down methods begin by dividing a sequence into segments in

a way that minimizes a cost function. In the E-Divisive method, implemented in the `ecp` R package (*15*) and considered a more sophisticated version of the binary segmentation method, the cost function involves the p-values from permutation tests that test whether the data from the proposed segments are sampled from different distributions. This is repeated for each resulting sub-sequence until a stopping criterion is met.

- *Optimal (PELT)* - optimal methods seek a sequence segmentation which directly minimizes a cost function related to the assumed likelihood function of the changepoint type, e.g., changes in mean, changes in variance, etc. The PELT (Pruned Exact Linear Time) method (*16*) solves the problem exactly with only linear computational cost, and is implemented in the `ruptures` Python package (*14*).

- *Bottom-up (Bottom-up)* Bottom-up methods begin with many initial changepoints and then merge the resulting subsequences until only a fixed (inputted) number of changepoints are remaining. In this analysis, the classical bottom-up algorithm as implemented in `ruptures` (*14*) is used.

- *Window-sliding (Window-sliding)* In window-sliding methods, a discrepancy measure (usually derived from a two-sample statistical test) is computed between a window and its previous location as it slides along a sequence, and then a peak-finding algorithm applied to the measures is used to detect changepoints. As with the bottom-up category, the classical method, explained in (*14*) and implemented in `ruptures`, is used in the present work.

Note that all four methods can be either univariate or multi-dimensional. The methods were applied both to each time series individually and to the multivariate time series.

In addition to aggregating results from multiple methods, each method was applied to the time series with multiple reasonable parameter settings to increase the strength of the analysis.

The parameter spaces for each method are outlined in Table S3. For each method, all parameter combinations ($2 \times 6 \times 5 = 60$) in the space were applied to the data, which yields 240 lists of changepoints.

For each possible changepoint location, the number of times the location appeared in a list was tallied. It can be assumed that the different methods will not identify the exact same changepoints; some deviation is expected. To account for this, tallies from consecutive or close changepoint locations were summed using the following rules:

- The tallies from two years may be aggregated if they are consecutive, e.g., 1965 and 1966, or two years apart. The resulting sum will be assigned to the earliest of the two years. For example, if 1965 and 1966 both have tallies of 60, aggregation will yield a tally of 120 for 1965 and 0 for 1966.

- The tallies from three different years may be aggregated if they are consecutive, e.g., 2007, 2008 and 2009. The resulting sum will be assigned to the median of the three years. For example, if 2007, 2008, and 2009 each have tallies of 20, aggregation will yield a tally of 60 for 2008 and 0 for 2007 and 2009.

- If the tallies from two years which are two years apart are being summed, the result will be assigned to the mean of the two years. For example, if 1969 and 1971 both have tallies of 30, aggregation will result in a tally of 60 for 1970 and 0 for 1969 and 1971.

The results are visualized in Figure S1.

After tallying, a threshold tally of 120 was set; that is, changepoints that appeared in at least half the function calls were considered "true" changepoints. From Figure S1, it is clear that Tonal Strength has a changepoint at 2000, PIC has changepoints at 1976 and 2000, Pitch SD has changepoints at 1976 and 2001, MIS has no changepoints, Onset Density has changepoints

at 1965 and 2000, TI-OD has a changepoint at 1995, ISO has a changepoint at 1997, RIC has a changepoint at 1996, and the multivariate time series has changepoints at 1975 and 2000.

With the univariate method, revolutions were defined as years that were identified as changepoints for at least two of the eight features. 1976 is a changepoint for two of the features, 1995, 1996 or 1997 are changepoints for three of the features, and 2000 or 20001 are changepoints for four of the features. Allowing for one year of deviation, this makes 1976, 1995-1997 (denoted 1996 hereafter) and 2000-2001 (denoted 2000 hereafter) the three melodic revolutions according to the univariate method.

The multivariate method selected the years 1975 and 2000 as changepoints, hence these are the revolutions yielded by the multivariate method. When comparing the revolutions from the two methods, it is reasonable to assume that revolutions that are one year apart are referring to the same period of change, so we consider the univariate method's 1976 changepoint and the multivariate method's 1975 changepoint to be equivalent. Therefore, 1975 and 2000 are the "strong", or Tier 1, revolutions, while 1996 is a Tier 2, or "moderate", revolution.

Autoregression and Regression of Residuals

The process to explore relationships between the time series was as follows:

1. *Autoregression* - autoregressive models were fit to each feature separately for each era using `AutoReg` in Python's `statsmodels` library, and the residuals were computed. As stated in the main text, there were maximum lags of 5, 5, and 4 imposed for Eras 1, 2 and 3, respectively. Within these limits, the lag yielding the best nRMSE was selected.

2. *Individual Regression* - for each era, each residual was regressed against every feature (except the feature from which the residual was computed) individually. Only regressions with R-squared values larger than 0.25 and p-values less than 0.05 were considered significant; the rest were discarded.

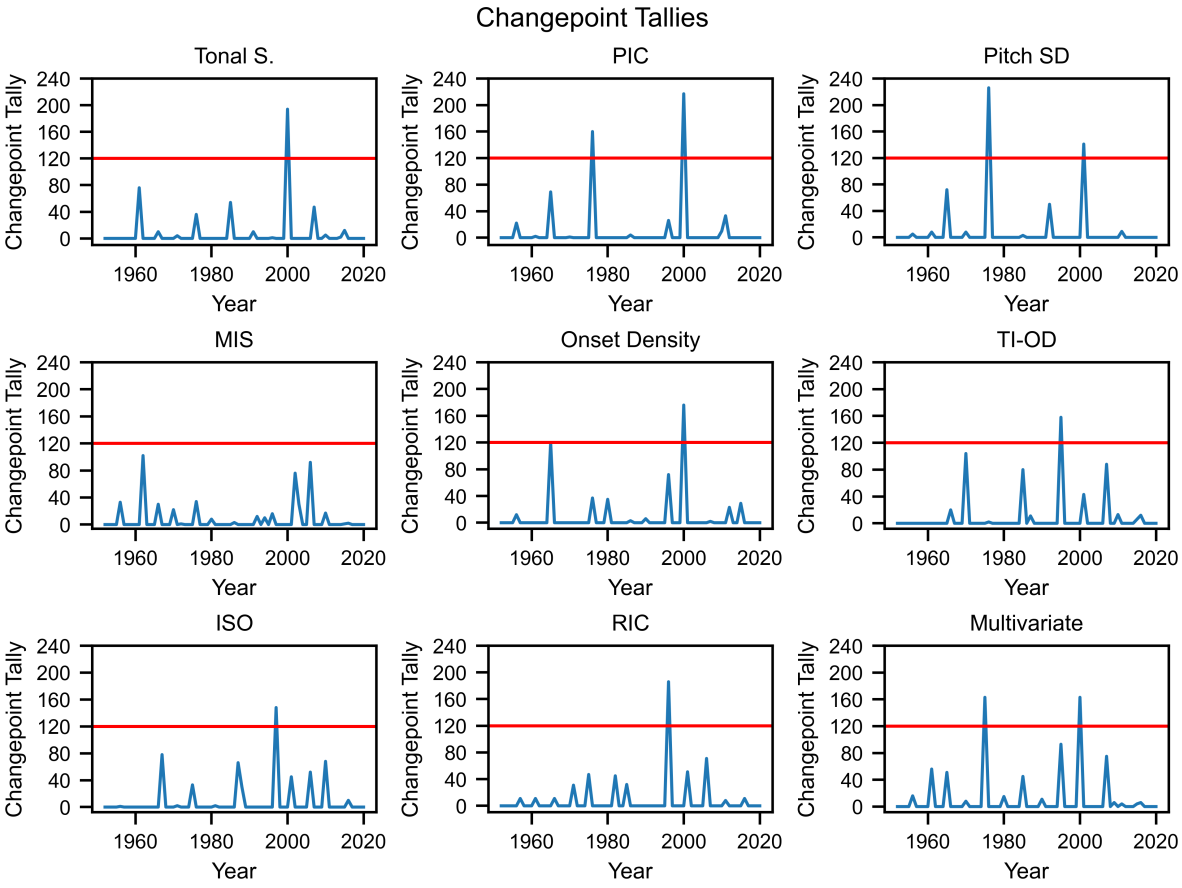

Figure 1: Tallies across 240 parameter combinations of four changepoint detection algorithms. The threshold tally, 120, is in red.

3. *Removing Collinearity for Multilinear Regression* - if a residual had more than one significant predictor, the correlations between these predictors were calculated. If two predictors were highly correlated ($r \geq 0.5$), the predictor with the lower R-squared value was discarded.

4. *Multilinear Regression* - if at least two uncorrelated predictors of a residual remained, multilinear regression was performed. In this study, since some features displayed high correlation within the eras, no multilinear regressions were performed.

Vector Autoregression

A separate VAR model was fit for each of the three eras. The modelling process was as follows:

1. *Granger's Causality Test* - vector autoregression assumes that the time series in the dataset influence each other. The null hypothesis of Granger's causality test is the negation of this assumption, that for the regression equation of one feature, the coefficients of the past values of other time series are zero. Granger's causality test was performed for every possible pair of features, which forms a matrix. Features whose associated row and column, in total, contained less than 2 p-values below .05 were discarded. No features were discarded for any era.

2. *Finding the Optimal Lag* - a vector autoregression model may include regression coefficients for time series values from one year ago, one year ago and two years ago, etc. To avoid overfitting, the optimal lag was selected by iteratively fitting VARs of higher and higher orders and calculating their Bayesion Information Criterion (BIC) until the BIC stopped decreasing. VAR(1) models were fit for all eras.

3. *Fitting the Model* - the VAR model of optimal order was fit to the data. Significant coefficients (p $\leq$ .05) from the models were used to calculate the fitted values shown in

Figure 2 in the main paper.

VAR Predictions for 2023

The third era VAR's forecasts for top melody feature values in 2023, as well as the observed feature values for 2023, are in Figure S2. The percent differences between the actual and forecasted values are reported in Table 4, as well as the root mean squared error, normalized by the standard deviation of the feature's time series from positions 2000 to 2022. Tonal Strength, Pitch SD and ISO are predicted to have upticks, while PIC, MIS, Onset Density, TI-OD and RIC are predicted to decrease. The VAR only predicts the correct direction for the ISO and RIC features, only half the predictions (Tonal Strength, Pitch SD, Onset Density and TI-OD) lie within a 10% error margin, and nRMSE is poor for all features except Tonal Strength, Onset Density and TI-OD. The mean percent error between the actual and forecasted values is 12.53, and the mean nRMSE is 2.04.

Cyclical Behavior in Pop Music History

Figure S3 visualizes the mean BPM of the top five songs per year, calculated from BiM-MuDa metadata (available on GitHub, see above). Smoothing is performed (two-forward, two-backward mean smoothing) to highlight cyclical behavior, which is mentioned in the Discussion section of the main paper.

References

1. C. Krumhansl, *Cognitive Foundations of Musical Pitch* (Oxford University Press, Oxford, 1990).

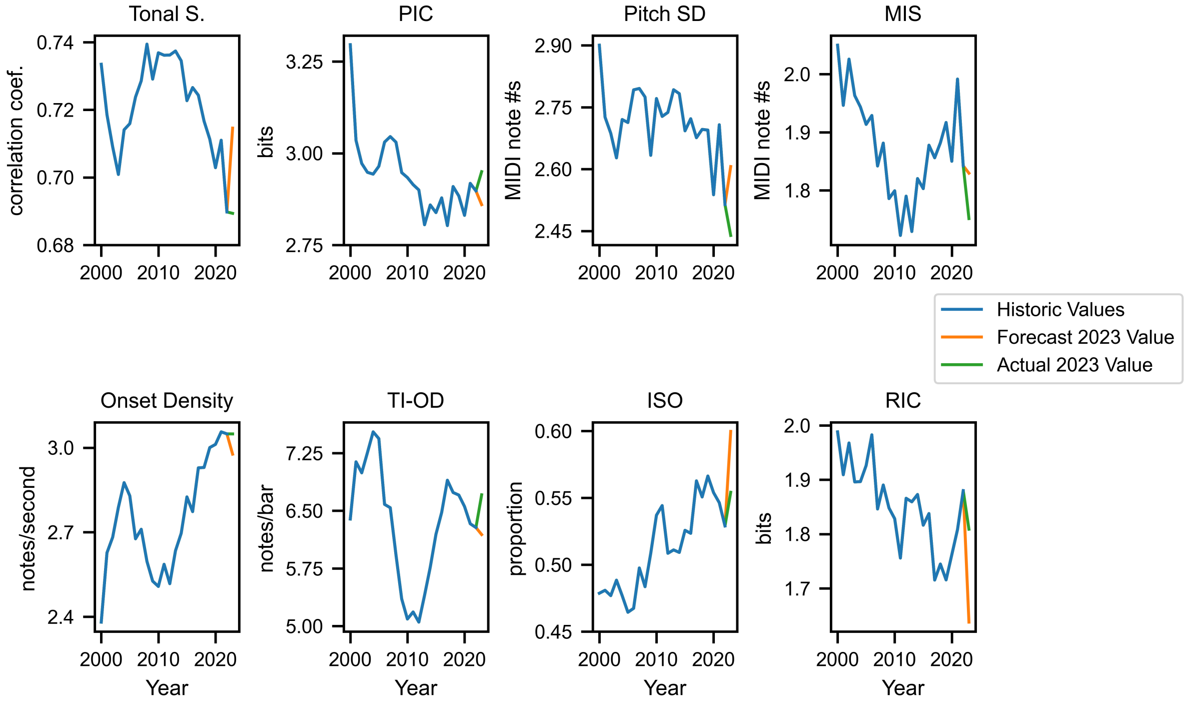

Figure 2: Time series values for all eight features in Era 3 (2000 - 2023), plus the forecast values for 2023 as predicted by the VAR fit to the Era 3 data. The time series are smoothed as in Figure 1 for time positions 2000 to 2020; values for the 2021-2023 positions are two-backwards smoothed, and 2023 forecast values are unprocessed.

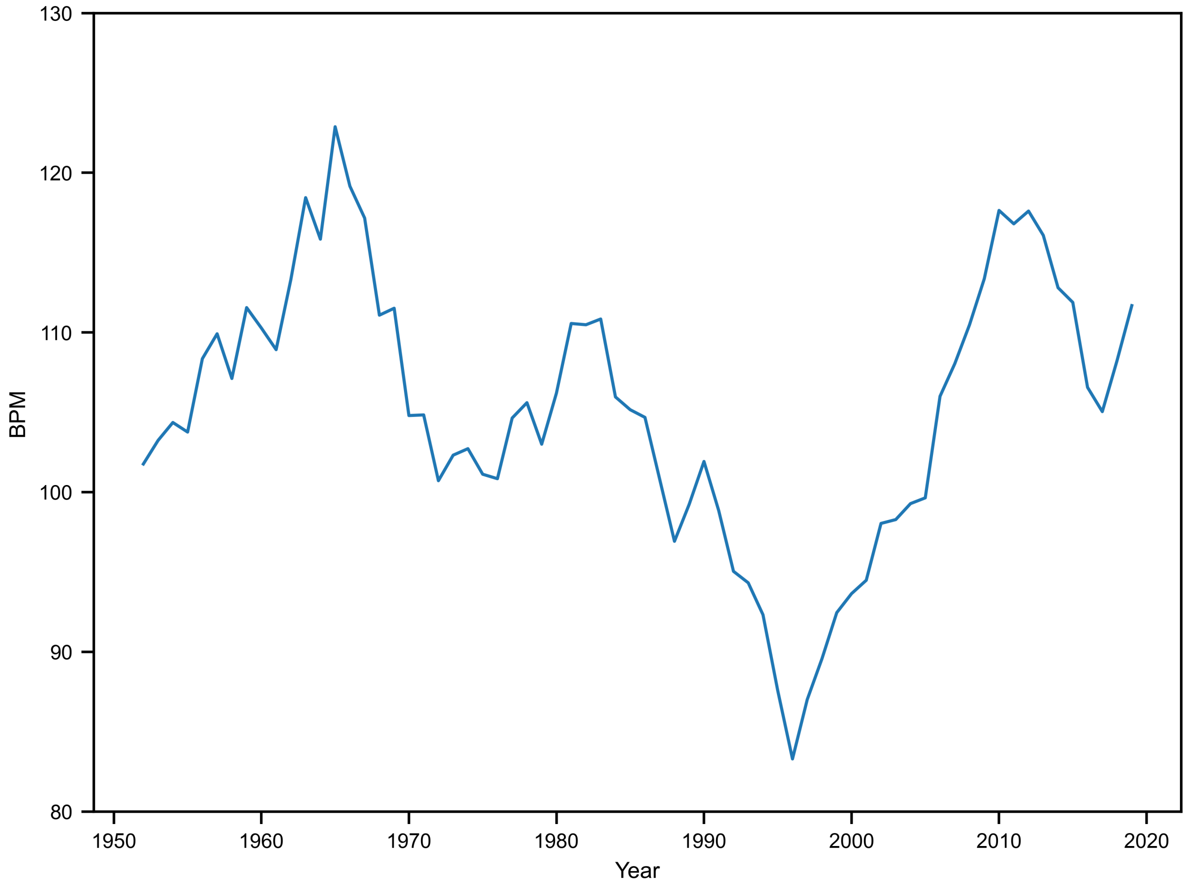

Figure 3: Mean BPM of the top five songs per year according to *Billboard*

2. M. Pearce, The construction and evaluation of statistical models of melodic structure in music perception and composition, Ph.D. thesis, School of Informatics, City University, London (2005).
3. N. Condit-Schultz, *Music Perception* **36**, 300 (2019).
4. M. Pearce, *Annals of the New York Academy of Sciences* **1423**, 378 (2018).
5. J. Cleary, I. Witten, *IEEE Transactions on Communications* **32**, 396 (1984).
6. S. Bunton, *The Computer Journal* **40**, 76 (1997).
7. T. Eerola, *Empirical Musicology Review* **11**, 2 (2016).
8. A. Clemente, *et al.*, *Behavior Research Methods* **52**, 1491 (2020).
9. A. Clemente, T. Kaplan, M. Pearce, *Annals of the New York Academy of Sciences* **1533**, 169 (2024).
10. S. Sauvé, M. Pearce, *Music Perception* **37**, 165 (2019).
11. T. Kaplan, Probabilistic models of rhythmic expectation synchronisation, Ph.D. thesis, School of Electronic Engineering and Computer Science, Queen Mary University of London, London (2024).
12. H. C. Longuet-Higgins, C. S. Lee, *Music Perception* **1**, 424 (1984).
13. A. D. Patel, J. R. Daniele, *Cognition* **87**, B35 (2003).
14. C. Truong, L. Oudre, N. Vayantis, *Signal Processing* **167** (2020).
15. N. James, W. Zhang, D. Matteson, ecp: Non-parametric multiple change-point analysis of multivariate data, https://cran.r-project.org/web/packages/ecp/index.html (2023).

16. R. Killick, P. Fearnhead, I. Eckley, *Journal of the American Statistical Association* **107** (2012).

Table 2: Rejected Feature Details

**Feature**	**Description**	**Reason for Exclusion**
PIC (LTM + STM)	The average information content of the pitches of a melody as computed by IDyOM, configured to predict the `pitch` viewpoint with the `interval` $\otimes$ `scale-degree` viewpoint using both the long-term and short-term models.	Correlates highly with PIC ($r = .92$), a melody's conformity with respect to other melodies, which would be measured by the LTM, is not the focus of this work.
Information Content (Pitch + Onset)	The average information content of a melody as computed by IDyOM, configured to predict the `pitch` and `onset` viewpoints with the `interval` $\otimes$ `scale-degree` and `ioi` viewpoints using the short-term models.	Unspecific; both pitch-related and rhythm-related complexity contribute to the IC values. We opted for PIC and RIC to decouple pitch-related and rhythm-related complexity.
Length (seconds)	The length of the melody in seconds	Onset Density captures more relevant information.
Length (notes)	The number of note events in the melody	Onset Density captures more relevant information.
Average Note Length	The average length of a note in the melody	Correlates highly with Onset Density ($r = -.90$), transcribed offset times of notes are generally less accurate than onset times.
Pitch Range	The difference between the highest and lowest MIDI note numbers in the melody	Correlates highly with Pitch SD ($r = .91$), and does not account for the spread of pitches in the melody.
Syncopation Index	The degree of syncopation in the melody according to Longuet-Higgins and Lee's model of syncopation (*12*)	The degree of syncopation can be thought of as a marker of rhythmic complexity, but the RIC feature is a more general measure. Additionally, the RIC feature provides a rhythmic counterpart to the PIC feature.
RIC (LTM + STM)	The average information content of the pitches of a melody as computed by IDyOM, configured to predict the `onset` viewpoint with the `position-in-bar` $\otimes$ `bar-length` viewpoint with both the short-term and long-term models.	A melody's conformity with respect to other melodies, which would be measured by the LTM, is not the focus of this work.
Normalized Pairwise Variability Index (nPVI)	durational constrast between consecutive onsets. Patel et. al (*13*) describe it as "the durational difference between each pair of intervals... measured relative to the average length of the pair".	Isochrony Proportion is more interpretable, captures the same variation nPVI captures, and is more relevant for music research (*3*).

Table 3: Changepoint Method Parameter Settings

Method	Parameter	Description	Subset Used
E-divisive	alpha	1 = search for changes in mean and variance, 2 = search for changes in mean only	$\{1, 2\}$
	min	minimum number of years between changepoints	$\{5, 6, 7, 8, 9, 10\}$
	k	number of changepoints to estimate; NULL returns all statistically significant changepoints	$\{NULL, 1, 2, 3, 4\}$
PELT	cost	cost function for optimization	$\{L1, L2\}$
	min	minimum number of years between changepoints	$\{5, 6, 7, 8, 9, 10\}$
	pen	parsimony penalty for model selection	$\{0.20, 0.65, 1.10, 1.55, 2.0\}$
Bottom-up	cost	cost function for optimization	$\{L1, L2\}$
	min	minimum number of years between changepoints	$\{5, 6, 7, 8, 9, 10\}$
	k	number of changepoints to estimate; for NULL, pen = log(n) = log(69) is used for an optimal value as suggested by (*14*)	$\{NULL, 1, 2, 3, 4\}$
Window-sliding	cost	cost function for optimization	$\{L1, L2\}$
	win	size of sliding window	$\{4, 6, 8, 10, 12, 14\}$
	k	number of changepoints to estimate; for NULL, pen = log(n) = log(69) is used as an optimal value	$\{NULL, 1, 2, 3, 4\}$

Table 4: Forecasting Errors for 2023 Time Series Values Produced by the Era 3 VAR

Feature	Percent Error	nRMSE
Tonal S.	-0.478	0.300
PIC	-14.8	4.64
Pitch SD	-6.06	2.30
MIS	13.2	2.50
Onset Density	5.31	0.889
TI-OD	-6.89	0.601
ISO	12.4	2.09
RIC	-12.8	3.03
